# Viewing second opinions in terms of recent developments in patient choice

**DOI:** 10.1186/2045-4015-1-31

**Published:** 2012-07-24

**Authors:** Richard B Saltman

**Affiliations:** 1 Department of Health Policy and Management, Rollins School of Public Health, Emory University, 1518 Clifton Rd NE, Atlanta, Georgia, 3033, USA

**Keywords:** Second opinion, Patient choice, Shared decision-making

## Abstract

Patient choice has become an increasingly visible part of publicly funded health care systems. Since the 1990s, many individuals have gained the ability to select their insurer in social health insurance funded systems, while in tax-funded health systems many patients can now select their primary care and hospital providers. Second opinions about clinical procedures are part of this broad movement toward increased patient involvement in care-related decision-making. One interesting policy question will be whether the coming period of financial austerity will strengthen or weaken the role of choice as health systems seek to deal with the inevitable mismatch of demand for and supply of medical resources.

## 

Seeking a second opinion [1] is part of a constellation of choices that patients are increasingly entitled to make about their health care services. In social health insurance funded systems, where patients have long had the ability to choose their primary care physician and hospital (although sometimes not hospital specialist), they have now become able to choose their insurer (Netherlands, Germany) [2,3]. In tax-funded health systems, depending on the country, patients have generally acquired the ability to choose their primary care facility and doctor if these are publicly run (Sweden) – while patients have long had at least some ability to choose their primary care doctor in systems where those physicians were private GPs (United Kingdom, Denmark) [4,5]. Similarly, these patients often now can choose their hospital in tax-funded systems, especially if they face a prolonged waiting period (although they usually cannot choose their hospital specialist) [6].

This complex framework of patient choice has grown considerably over the past 25 years. A combination of better informed patients and, in some systems, substantial waiting lists have pushed policymakers to open up what previously had been an expert-determined planning process. Planners’ concerns that allowing choice would cost more (due to extra capacity) as well as reduce overall equity have largely been replaced by politicians’ concerns that they not be blamed by increasingly vocal patients for inadequately responsive provider institutions.

This growth in patient choice also represents a desire of many patients to participate in their own medical decision-making [7]. A whole branch of medical research and decision-making has grown up devoted to assisting patients in helping make clinical decisions that have social and lifestyle consequences such as those dealing with breast and prostate cancers. See, for example, the work of the Informed Medical Decisions Foundation in Boston [8]. The Picker Institute in England has published numerous studies on how patients can be more involved in care-related decisions [9].

One interesting issue is how well this entire apparatus of patient choice –potentially including second opinion– will sustain itself as health systems confront what is shaping up to be a new era of permanent austerity [10]. While the principles of patient choice are fairly well embedded in many health systems, tax-funded health systems in particular may face pressures to cut back aspects of choice that are perceived to require additional expenditure and/or personnel time. In this regard, although second opinion has the advantage of reducing costs from unnecessary procedures, some public policymakers may seek to trim down the numbers of second opinions to only those interventions and/or cases where past research has shown substantial likelihood of cost savings.

Patients, conversely, may well push for increased choice options, especially if the austerity process results in making patients contribute more out-of-pocket to the overall cost of the procedures they receive. If they have to pay more, they may reason, they ought to have more of a say in deciding what should be done. This may especially apply to second opinions for expensive and invasive clinical procedures. Increased patient expectation of participation in medical decision-making may also develop if some tax-funded health systems, facing serious funding shortfalls, turn to private providers for particular procedures where private provision (especially if they do not involve union contracts) may be cheaper.

## Competing interests

Dr. Saltman has no commercial conflicts of interest.

## Author information

Richard B. Saltman is Professor of Health Policy and Management at the Rollins School of Public Health at Emory University in Atlanta. He is also Associate Director of Research Policy at the European Observatory on Health Systems and Policies in Brussels.

Commentary on the paper by Greenfield, Pliskin, Wientroub, Davidovitch: Orthopedic surgeons’ and neurologists’ attitudes towards second opinions in the Israeli healthcare system: a qualitative study.
